# Radiological imaging of pericardial hydatid cyst

**DOI:** 10.1590/0037-8682-0753-2020

**Published:** 2021-03-08

**Authors:** Okan Cakir, Recep Sade, Fatih Alper

**Affiliations:** 1 Ataturk University, Medical Faculty, Department of Radiology, Erzurum, Turkey.

**FIGURE 1:**
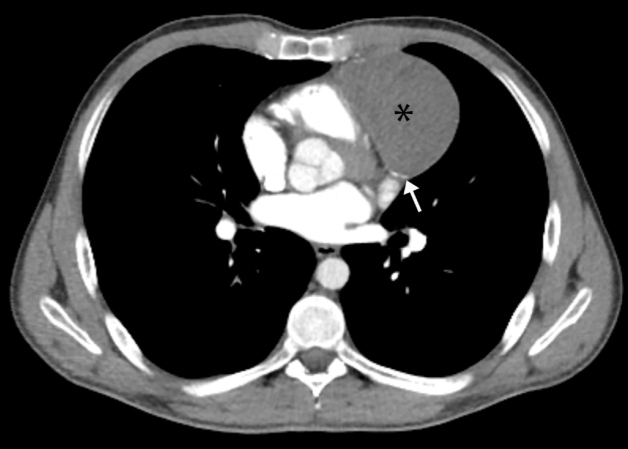
Axial contrast-enhanced computed tomography. There is a pericardial heterogeneous cystic mass (asterisk) containing peripherally located foci of calcifications (arrow).

**FIGURE 2:**
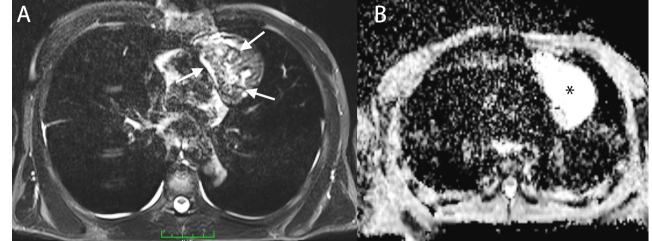
T2-weighted **(A)** and diffusion-weighted imaging **(B)** showing a pericardial mass that is heterogeneous isohyperintense with a hypointense wall (arrow). There was no restriction of diffusion on diffusion-weighted imaging (asterisk).

A 28-year-old man presented with chest pain. Laboratory investigations revealed mild leukocytosis with eosinophilia. Multidetector computed tomography (MDCT) revealed a pericardial heterogeneous cystic mass containing peripherally located foci of calcifications (Figure 1, white arrow) and no contrast enhancement (Figure 1, asterisk). T2-weighted magnetic resonance imaging (MRI) revealed a pericardial mass, which was heterogeneous isohyperintense with a hypointense wall (Figure 2A, arrow). The mass was heterogeneously isointense on contrast-enhanced T1-weighted MRI. There was no restriction of diffusion on diffusion-weighted imaging (Figure 2B, asterisk). Therefore, the patient was diagnosed with a pericardial hydatid cyst.

Hydatid disease manifests as a hydatid cyst^1^ ^,^ ^2^ that most commonly occurs in the liver and lungs. The tapeworm commonly involved is *Echinococcus granulosus* ^1^ ^,^ ^2^. Cardiac hydatid cysts make up 0.5%-2% of all cases and are usually situated in the ventricles or rarely in the pericardium^1^. Transthoracic echocardiography, MDCT, and MRI can show the mass’s cystic nature and its relationship to the cardiac chambers. Transthoracic echocardiography may be inadequate to define the cyst and its relationship to adjacent structures. MDCT and MRI can be used to evaluate cysts more accurately.

In conclusion, pericardial hydatid cyst is a rare condition that should be kept in mind, especially in patients from endemic areas.
